# mTORC1 inhibition by sirolimus as adjunctive treatment in experimental pneumococcal meningitis

**DOI:** 10.1093/braincomms/fcaf460

**Published:** 2025-11-25

**Authors:** Rutger Koning, Dixie Bakker, Marian A van Roon, Valery Jaspers, Matthijs C Brouwer, Diederik van de Beek

**Affiliations:** Department of Neurology, Amsterdam UMC, University of Amsterdam, Amsterdam Neuroscience, 1105 AZ Amsterdam, the Netherlands; Department of Neurology, Amsterdam UMC, University of Amsterdam, Amsterdam Neuroscience, 1105 AZ Amsterdam, the Netherlands; Department of Neurology, Amsterdam UMC, University of Amsterdam, Amsterdam Neuroscience, 1105 AZ Amsterdam, the Netherlands; Department of Neurology, Amsterdam UMC, University of Amsterdam, Amsterdam Neuroscience, 1105 AZ Amsterdam, the Netherlands; Department of Neurology, Amsterdam UMC, University of Amsterdam, Amsterdam Neuroscience, 1105 AZ Amsterdam, the Netherlands; Department of Neurology, Amsterdam UMC, University of Amsterdam, Amsterdam Neuroscience, 1105 AZ Amsterdam, the Netherlands

**Keywords:** bacterial meningitis, *Streptococcus pneumoniae*, experimental meningitis, sirolimus, mTORC1

## Abstract

Sirolimus, an inhibitor of mammalian target of rapamycin complex (mTORC) 1, a regulatory protein involved in seizures and a modulator of inflammation, represents a potential new treatment strategy for pneumococcal meningitis. This study investigates the effects of mTORC1 inhibition using sirolimus in a mouse model of experimental pneumococcal meningitis. In a prospective, investigator-blinded and randomized trial, 96 mice were infected intracisternally with *Streptococcus pneumoniae* serotype 2. Mice were randomized for treatment with sirolimus and all mice received antibiotic treatment. In a clinical severity experiment, 48 mice were infected and scored every 4 h for disease severity until 72 h. In a time-point experiment, 48 mice were infected and terminated at 6 or 24 h after infection for evaluation. In the clinical severity experiment, sirolimus treatment did not improve survival, although seizures tended to occur less in treated mice [3 of 24 (13%) versus 5 of 24 (21%), *P* = 0.7]. In the time point experiment, clinical severity scores were increased in sirolimus-treated mice [maximum difference at 24 h after infection with a median of 10 (interquartile range 10–13) versus 7.5 (interquartile range 5–9) at 24 h after infection, *P* < 0.01]. While bacterial loads were similar across groups, sirolimus treatment increased inflammation. In the brain of treated mice, interleukin 6 was increased (median 8900 pg/ml versus 4804 pg/ml, *P* = 0.04), while interleukin 12 was elevated in both the brain (median 591 pg/ml versus 405 pg/ml, *P* = 0.03) and plasma (median 216 pg/ml versus 7 pg/ml, *P* < 0.01). Our findings illustrate the importance of mTORC1 signalling during pneumococcal meningitis in regulating inflammation. However, our results indicate that sirolimus is unlikely to be an effective treatment for this condition.

## Teaser

Sirolimus, an mTORC1 inhibitor, was tested as adjunctive treatment in a mouse model of pneumococcal meningitis. Sirolimus worsened clinical severity and increased inflammation, elevating IL-6 and IL-12 levels. Findings highlight mTORC1’s role in inflammation but suggest sirolimus is ineffective for treating pneumococcal meningitis.

## Graphical Abstract

**Graphical Abstract:**
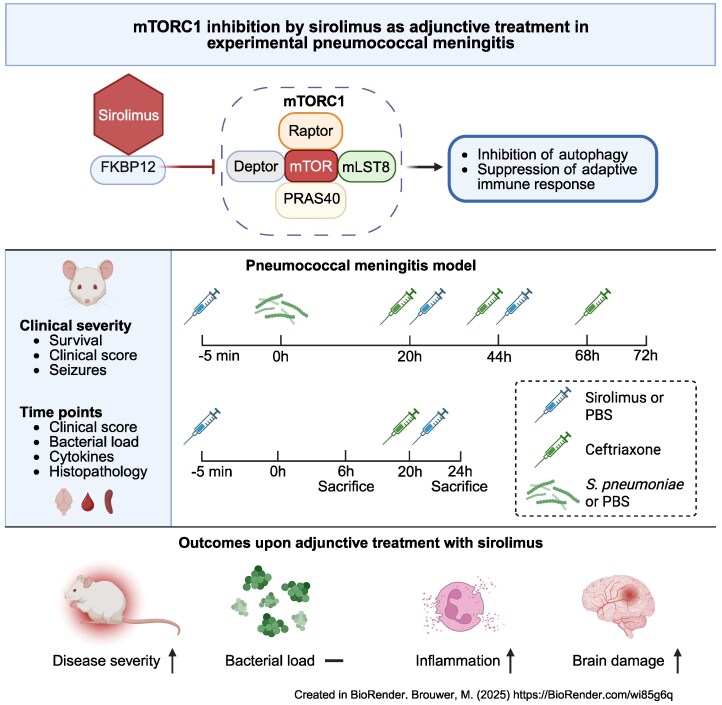

## Introduction

Bacterial meningitis is a severe infection of the central nervous system, mostly caused by *Streptococcus pneumoniae.*^1,2^ Mortality rates range from 10% to 35%, and half of survivors suffer from neurological sequelae such as focal neurological deficits, hearing loss and cognitive impairment.^3-5^ Seizures occur in patients at high rates (17%) and are associated with mortality.^6^ Adjunctive treatment with dexamethasone, an anti-inflammatory drug, has improved outcomes in recent years, but there remains a need for more effective (adjunctive) treatments.^7-9^

Human genetic variation explains almost half of the variation in susceptibility to pneumococcal meningitis and one-third of variation in severity.^10^ A genome-wide association study found an association between a genetic variant in the AKT serine/threonine kinase (AKT) 3 gene and outcome in patients with pneumococcal meningitis, which was mainly driven by seizure activity.^11^ AKT3 is highly expressed in the central nervous system, and mutations in the phosphoinositide 3-kinase (PI3K)-AKT- mechanistic target of rapamycin (mTOR) pathway lead to epilepsy through mTOR hyperactivation, a group of disorders called mTORopathies.^12,13^

mTOR is a regulatory protein involved in cellular homeostasis and metabolism, response to growth factors, inflammation and autophagy.^14-17^ mTOR forms two distinct complexes, mTOR complex (mTORC) 1 and mTORC2, by combination with protein binding partners, of which mTORC1 is modulated through the AKT pathway and can be pharmacologically inhibited by sirolimus.^18^ In epileptic patients with tuberous sclerosis complex (TSC), a genetic disease characterized by hyperactivation of mTOR, clinical trials showed that sirolimus treatment reduced the incidence of seizures.^19-22^ Everolimus, another inhibitor of mTORC1, has been approved for the treatment of epilepsy in TSC patients.^23^ mTORC1 is also a regulator of inflammation and its inhibition by sirolimus is used to prevent organ rejection after kidney transplantation.^24,25^ mTORC1 has pleiotropic effects on inflammation through metabolism, cell migration and autophagy and inhibition can lead to both pro-or anti-inflammatory effects depending on cell type, inflammatory stimulus and timing.^24,26^

This study explores whether mTORC1 inhibition by sirolimus improves outcomes in murine pneumococcal meningitis. Sirolimus was administered as a pre-treatment in a mouse model of pneumococcal meningitis, and its effects were assessed on clinical severity, bacterial load and immune response.

## Materials and methods

Experiments were conducted using 108, 8–12-week-old immune competent female and male C57BL/6N mice obtained from Charles River Laboratories. Mice were housed in individually ventilated cages with corncob bedding under controlled conditions. Animal experiments were authorized by the Centrale Commissie Dierproeven Netherland (document no. AVD11800202013797) and the local ethics committee ‘DEC-AMC’ (document no. NEU20-13797-1-04 and -05). Experiments followed institutional guidelines and Animal Research: Reporting of *In* Vivo Experiments (ARRIVE) reporting standards.

A well-characterized mouse model of pneumococcal meningitis was used.^27-29^ Mice were acclimatized for 1 week prior to experiments and randomized into treatment groups using RANDOM.ORG. Researchers were blinded for treatment during experiments and data analysis. At the start of the experiment (Day 0), mice were weighed and clinically scored based on weight loss, activity, posture, skin/fur condition, eye appearance, breathing, seizures, limb paresis or ataxia (Supplementary Table 1). Animals reaching humane endpoint (HEP) criteria (clinical scoring ≥14, weight loss ≥25%, coma, paralysis, seizure longer than 5 min or two seizures in 15 min.) were euthanized. Mice were inoculated intracisternally with 1 µl of bacterial suspension containing *S. pneumoniae* serotype 2 (D39) or phosphate-buffered saline (PBS) into the cisterna magna under isoflurane anaesthesia. Neurological damage from the inoculation was assessed using the Foot Fault Grid test, and affected mice were excluded.

Sirolimus (5 mg/kg) or vehicle (distilled water containing 5% dimethyl sulfoxide, 5% polyethylene glycol 400% and 5% polysorbate 80) was administrated intraperitoneally 5 min before inoculation and at 20 and 44 h post infection (hpi). Ceftriaxone (100 mg/kg bodyweight) was given intraperitoneally at 20 hpi and every 24 h thereafter. Mice were clinically scored every 4 h from 16 hpi onwards until the endpoint or HEP. Mice reaching HEP were scored as 15 points for the remainder of the study. At the experimental endpoint of the study mice were anaesthetized by intraperitoneal injection of 190 mg/kg ketamine and 0.3 mg/kg dexmedetomidine in a total volume of 300 µl, followed by cardiac puncture for blood collection and perfusion of organs with sterile PBS via the left ventricle. Cerebrospinal fluid (CSF) was collected by puncture of the cisterna magna. Left hemisphere of brain, lung and spleen were harvested, weighed and placed in 4× volume weight of ice-cold 0.9% NaCl solution, processed and stored as described before.^28^ Ethylenediaminetetraacetic acid blood was centrifuged at 2000*g* for 15 min. Plasma was stored at −80°C until further analysis.

For the clinical severity experiment the following groups were defined: (i) adjunctive sirolimus group receiving sirolimus: *n* = 24 (12 female) and (ii) control treatment group receiving vehicle: *n* = 24 (12 female). We aimed to detect a difference in clinical severity score of 2.5 points. Using an 80% power, two-sided testing, and significance level of *P* < 0.05, we needed 24 mice per group. Mice were infected with 1.35 × 10^6^ colony-forming units (CFU) *S. pneumoniae* and were clinically scored every 4 h for 72 h.

For the time point experiments, 60 mice were randomly distributed over the following experimental groups: (i) uninfected/standard treatment group (*n* = 12, six female) mock infected with PBS, mock treatment with vehicle; (ii) infected/standard treatment group (*n* = 24, 12 female) infected with 0.37 × 10^6^ CFU *S. pneumoniae*, mock treatment with vehicle and (iii) infected/sirolimus treatment group (*n* = 24, 12 female) infected with 0.37 × 10^6^ CFU *S. pneumoniae*, sirolimus treatment (5 mg/kg). Load of infection was set to 0.37 × 10^6^ CFU for the time point experiments compared to 1.35 × 10^6^ for the clinical severity experiment to maximize the chance of affected animals and at the same time minimize the chance of death of the animals in the chosen treatment interval. We aimed to detect a difference in inflammatory parameters with an effect size (δ = |µ₁-µ₂|/σ) of 1.3. Using 80% power, two-sided testing, and a significance level of *P* < 0.05, we needed 12 mice per group. Half of the mice in each group were euthanized at 6 hpi, the other half at 24 hpi. Experiments were performed in two sessions of 24 mice each, with equal representation of all experimental groups in both sessions. At the experimental end points mice were euthanized, and their organs were processed and analysed. Outcome measures included clinical scores, CFU counts, cytokine concentrations, histopathological examination and gene expression in CSF, brain, spleen and plasma.

CFU counts were assessed by plating 50 µl undiluted plasma, CSF (diluted 1:100 in 0.9% NaCl) and homogenates of brain and spleen onto blood agar plates. Serial 10-fold dilutions (10^−1^ till 10^−7^) were prepared, and 10 µl of each dilution was plated. Plates were dried and incubated overnight at 37°C/5% CO_2_, and colonies were counted the following day. The bacterial inoculum into the cisterna magna was verified using the same method immediately before and after administration.

Murine cytokine levels [interleukin (IL)-1β, IL-6, IL-10, IL-12, interferon (IFN)-γ and tumour necrosis factor (TNF)] were measured in plasma and brain homogenates using BioPlex Pro Mouse Cytokine Assay (Bio-Rad Laboratories, item# 12019303) and a Luminex 200 reader. For the brain homogenates, the Standard Diluent from the Luminex assay kit was replaced by Brain Matrix Diluent (5% bovine serum albumin: 0,9% NaCl: Greenburg lysis buffer [1:4:5] + protease inhibitor mix) for diluting the brain homogenate samples as well as the Standard Dilution Series to minimize aggregation of the magnetic beads. Plasma and brain homogenate samples were diluted 4× and 2×, respectively, before analysis.

Histopathology was performed on the right hemisphere of the brain. Brain was fixed in 4% paraformaldehyde and paraffin-embedded in seven coronal plaques which were cut in sections of 5 μm. Samples were stained with haematoxylin-eosin (HE) with the Ventana BenchMark ULTRA system (Roche). Immunostaining was performed with antibodies against CD45 (Biolegend) to detect leukocytes followed by a haematoxylin counterstain. Histopathology was scored (blinded) by two researchers separately as previously described.^30^ A detailed description of the histological scoring method can be found in Supplementary Table 2. Total pathology score was calculated by adding individual scores for all categories together.

Total RNA was isolated from 50 µl of brain and spleen homogenate using the NucleoSpin RNA, mini kit for RNA purification (Macherey-Nagel, item# 740984). Complementary DNA (cDNA) was synthesized using the iScript cDNA Synthesis Kit (Bio-Rad). The real-time polymerase chain reaction (RT-PCR) measurement of individual cDNAs was performed on a Bio-Rad MyiQ Single-Color RT-PCR Detection System using the Bio-Rad iQ SYBR Green Supermix (Bio-Rad Laboratories). Gene expression of *Akt3* (FW primer CATCTGAAACAGACACCCGATA, RV primer GTCCGCTTGCAGAGTAGGAG), *IL-10* (FW primer AGCCTTATCGGAAATGATCCAG, RV primer GGCCTTGTAGACACCTTGGT) and *IL-12* (FW primer CTGGAGCACTCCCCATTCCTA, RV primer GCAGACATTCCCGCCTTTG) was determined in brain and spleen. Expression data were normalized to the *Nono* (FW primer AGGCTCCTTCTTGCTGACTAC, RV primer TCTCTCTCCTTGTGGAATTGCTG) and *Actb* reference genes and were analysed using the Bio-Rad MyiQ Optical system Software version 1.0.

Phosporylation of ribosomal protein S6 kinase B1 (p70 S6 kinase) was visualized with western blotting. In short, protein concentrations of brain homogenates from the 6 h timepoint were determined with the BCA Protein Assay (ThermoFisher, Cat). SDS-polyacrylamide gels (Bio-Rad) were used to separate 50 µg of protein, followed by transfer to PVDF membranes (ThermoFisher). Membranes were blocked in TBS-T + 5% bovine serum albumin for 1 h at room temperature and subsequently incubated with primary antibodies against p70 S6 kinase (1:750, Cell Signaling Technology, 9202), phospho-p70 S6 kinase (1:500, Cell Signaling Technology, 9234) or glyceraldehyde 3-phosphate dehydrogenase (1:5000, Bio-Connect, M00227-1). Overnight incubation was performed at 4°C. Next, membranes were washed and incubated with an HRP-conjugated secondary antibody (1:5000, ThermoFisher, G-21234), followed by visualization with an ECL detection system (ThermoFisher, Cat: 32106X4). Images were acquired using the Platinum V10 (Uvitech).

### Statistical analysis

After D’Agostino-Pearson normality testing, continuous variables were compared using either *t*-test or the Mann–Whitney *U* test, proportions were compared using Fisher’s exact test, and survival was analysed using the log rank test. For all analyses, a *P*-value of <0.05 was considered significant. Statistical analyses were conducted using Graphpad version 10.2.0.

## Results

**Figure 1:**
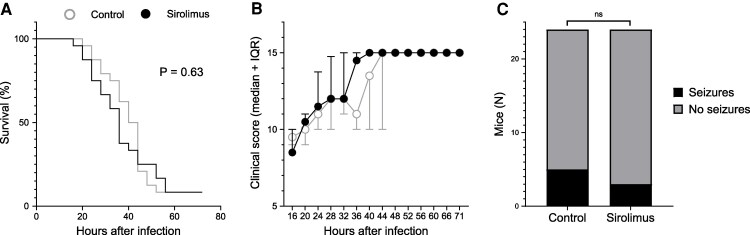
**Clinical severity experiment.** Sirolimus treatment did not improve outcome in the clinical severity experiment. Mice were infected a high bacterial load (1.35 × 10^6^ CFU *S. pneumoniae*). **(A)** Survival over 72 h was similar between treatment groups. **(B)** Clinical scores were not significantly different between sirolimus and vehicle treatment mice at any time point. The maximum difference in clinical scores was found at 36 hpi with a median score of 14.5 in the sirolimus-treated group compared to 11 in the control group (*P* = 0.09, U = 208.5). **(C)** Seizures tended to occur less in sirolimus-treated mice, although the difference was not significant. For **A–C**; *N* = 24 mice per group. Data are median ± IQR. Survival was compared using a Log-rank test; clinical scores were compared at every time point separately using a Mann–Whitney *U* test; seizure occurrence was compared using a Fisher’s exact test. ns, not significant.

For the clinical survival experiment, mice were intracisternally infected with a high bacterial load (1.35 × 10^6^ CFU *S. pneumoniae*). Baseline weights were comparable between treatment groups {21.7 g [interquartile range (IQR) 20.7–24.8] controls and 22.8 g [21.5–24.5] sirolimus; *P* = 0.52}. Across both treatment groups, 22 of 24 (92%) mice reached a humane end point within 72 h (Fig. 1). Median time until reaching the humane endpoint between both treatment groups was also similar (median survival 42 h for controls versus 36 h for the sirolimus group; *P* = 0.30). The maximum difference in clinical score was found at 36 h after infection [11.9 points (IQR 10–14.7) controls and 14.5 points (IQR 10–15) sirolimus, *P* = 0.09, Fig. 1].

Seizures were observed in 8 of 48 (17%) mice, including seven (88%) females. The first seizure was observed within 24 h after infection in six of eight (75%) mice, 32 h for one mouse, and one mouse developed status epilepticus at 48 h and therefore reached a humane endpoint. Seizures were observed only once in most mice [six out of eight (75%)], while two seizures were observed in one mouse and three in another. The rate of seizures was similar between sirolimus and control groups: 5 of 24 (21%) mice in the control group developed seizures and 3 of 24 (13%) in the sirolimus group (odds ratio 0.54, 95% confidence interval 0.13–2.59, *P* = 0.70, Fig. 1).

**Figure 2:**
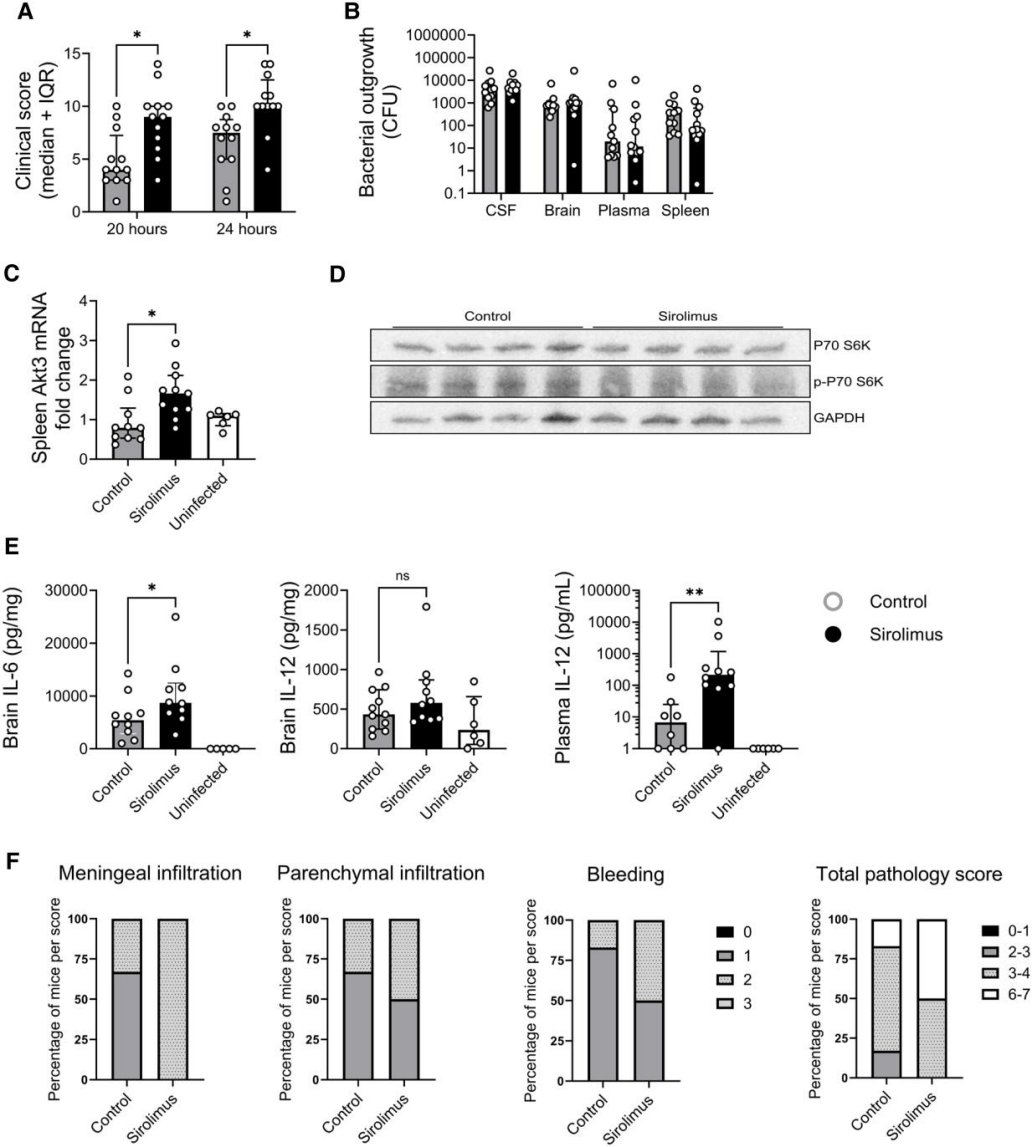
**Time point experiment.** In the time point experiment sirolimus treatment increased clinical scores and inflammation. Mice were intracisternally infected with a low bacterial load (0.37 × 10^6^ CFU *S. pneumoniae*). **(A)** Clinical scores at 20 and 24 h after infection were increased in sirolimus-treated mice. **(B)** Bacterial outgrowth in the CSF, brain, plasma and spleen at 6 h after infection was similar between treatment groups. **(C)**  *Akt3* mRNA, expressed as fold change, was increased in the spleen of sirolimus-treated mice at 24 h after infection. (**D**) Phosphorylation of P70 S6 kinase (p-P70 S6K) was decreased in the brain of mice treated with sirolimus at 6 h post treatment. **(E)** IL-6 and IL-12 concentrations were increased in the brain and plasma of sirolimus-treated mice at 24 h after infection. **(F)** Histological scores for meningeal and parenchymal infiltration, ventriculitis and bleeding of 4 sirolimus-treated infected mice compared to 6 vehicle-treated infected mice at 24 h after infection, expressed as percentage of mice per score, show more pathology in the sirolimus-treated group compared to the vehicle-treated group. For **A-C**, **E**; *N* = 12 per group for infected mice and *N* = 5–6 for uninfected controls. Each data point represents a measurement from an individual mouse. For statistical testing sirolimus-treated infected mice were compared to vehicle-treated infected mice (controls). Data are median ± IQR, D’Agostino–Pearson normality was performed and a Student’s *t*-test (**A**) or Mann–Whitney *U* test (**B, C, E**) was used for statistical analysis. *P < 0.05, **P = 0.0067. For **D**; *N* = 4 mice per group. The images of the blots were cropped. Full images can be found in Supplementary Fig. 1. Akt3, Akt Serine/Threonine Kinase 3; GAPDH, glyceraldehyde 3-phosphate dehydrogenase; mRNA, messenger ribonucleic acid; P70 S6K, ribosomal protein S6 kinase B1; p-P70 S6K, phosphorylated ribosomal protein S6 kinase B1.

For the time point experiment, mice were intracisternally infected with a low bacterial load (0.37 × 10^6^ CFU *S. pneumoniae*). One vehicle-treated mouse was taken out of the experiment due to a paresis directly after inoculation (6-h group). Baseline weights were similar between treatment groups [22.5 g (IQR 20.1–26.3) controls and 22.2 g (IQR 20.5–27.3) sirolimus, *P* = 0.68]. Of the sirolimus-treated mice, 2 of 12 (17%) reached a humane endpoint before 24 h, while none of the vehicle control mice did. Clinical scores were higher in the sirolimus-treated mice compared to controls at both 20 and 24 h (Fig. 2A). At 20 h, the median score of control mice was 4.0 (IQR 3–7.3) versus 8.7 (IQR 6.3–10.0) for sirolimus-treated mice (*P* = 0.007). At 24 h, the scores were 7.5 (IQR 5–8.8) for controls and 10.0 (IQR 10.0–12.5) for sirolimus (*P* = 0.002). No seizures were observed during time point experiments.

Bacterial counts (CFU) were measured in the brain, blood and spleen of mice at 6 and at 24 h. At 6 h, pneumococci were found in all compartments of infected mice, with no differences between treatment groups (Fig. 2B). At 24 h, CFUs counts decreased markedly in all compartments following antibiotic treatment, without differences between treatment groups.

Gene expression of *Akt3, IL-10* and *IL-12* in the spleen and brain revealed a modest increase in Akt3 expression in the spleen of sirolimus-treated mice at 24 h [fold change 1.7 (IQR 1.26–2.12) sirolimus and 0.80 (IQR 0.53–1.29) vehicle, *P* = 0.01, Fig. 2C]. Western blot analysis showed decreased presence of p-P70 S6 K in the brain of mice treated with sirolimus at 6 h, indicating that sirolimus effectively inhibited mTORC1 activation in this model (Fig. 2D). No differences were found in *IL-10* or *IL-12* gene expression between treatment groups in either the brain or spleen.

Cytokine levels (IFN-γ, IL-1β, IL-6, IL-10, IL-12, TNF) were measured in brain homogenates and the plasma. At 6 h, IL-6 was elevated in infected mice compared to noninfected animals, but there were no differences between treatments groups. At 24 h, sirolimus-treated mice had significantly higher IL-6 (median 8900 pg/ml versus 4804 pg/ml, *P* = 0.04) in the brain compared to vehicle-treated mice (Fig. 2E). In plasma, IL-12 was increased in sirolimus-treated mice (median 216 pg/ml versus 7 pg/ml, *P* < 0.01), while other cytokines showed no differences between groups (Fig. 2E).

Blinded histopathological analysis of brain slices of six vehicle-treated infected (control) and four sirolimus-treated infected mice at 24 h showed a higher degree of meningeal infiltration by CD45 + immune cells and slightly higher degrees of parenchymal infiltration, bleeding and ventriculitis comparing vehicle-treated mice to sirolimus-treated mice. Although numbers are too low for a significant statistical analysis, total pathology score is more severe in the sirolimus-treated mice (Fig. 2F), indicating an enhanced inflammatory response.

## Discussion

This study highlights the role of mTORC1 signalling in regulating the inflammatory response during pneumococcal meningitis despite comparable bacterial loads between groups. Sirolimus-treated mice exhibited higher clinical severity scores, more severe histopathology, and an amplified inflammatory response, suggesting that mTORC1 activity may be essential for maintaining immune balance and limiting pathological inflammation in the CNS during pneumococcal meningitis.

While sirolimus is recognized for its immunosuppressive properties, particularly in the context of T-cell-mediated adaptive immunity, its effects on innate immune responses are more complex and context dependent. In our model, sirolimus treatment led to elevated levels of the inflammatory cytokines IL-6 and IL-12 and an enhanced inflammatory response by recruitment of immune cells. These findings are consistent with prior studies, indicating that mTORC1 inhibition can have pro-inflammatory effects, such as increasing the release of cytokines including IL-12 and IL-23, while suppressing anti-inflammatory IL-10 production.^31-35^ mTORC1 inhibition may prolong acute inflammation by impairing the resolution phase, disrupting the normal transition from neutrophil to monocyte recruitment.^24,36^ Together, these mechanisms may underlie the heightened inflammation and worsening clinical outcomes observed in sirolimus-treated animals.

Our findings contrast with previous studies in other infectious disease models, where sirolimus pre-treatment improved survival or reduced pathology. A meta-analysis of experimental studies showed that 13 of 29 (45%) studies reported improved survival by sirolimus (pre)treatment in systemic and respiratory infections with viral, parasitic, fungal or bacterial pathogens.^37^ Similarly, in a murine pneumococcal pneumonia model, sirolimus pre-treatment increased survival and reduced lung damage, without changing lung bacterial loads or cytokine concentration.^37,38^ In this study, a different mouse strain was used (UM-HET2), mice were older (24 months), had been pre-treated with sirolimus for 17 weeks and mice were infected with a different pneumococcal serotype (serotype 4, TIGR4). These factors highlight the importance of infection site, host background, timing and disease phase when interpreting the effects of mTORC1 inhibition.

The timing of sirolimus administration in our study, initiated shortly before infection, may also have influenced its effects. mTORC1 inhibition can modulate leukocyte recruitment, but the short pre-treatment window might have been insufficient to affect early cellular migration. Conversely, later administration, during the peak inflammatory phase, could have yielded different outcomes. This underscores the dynamic and stage-specific nature of mTORC1 signalling in CNS infections.

Our study had limitations. Typically, mTORC1 inhibition suppresses neutrophil and macrophage migration that would be expected to result in decreased inflammation in our model.^24,39,40^ Sirolimus was administered only a short period prior to disease onset, potentially missing the opportunity to inhibit early leukocyte migration. Treatment at a later time point could have resulted in a different effect of sirolimus treatment considering the complex interplay of mTORC1 activation and inflammation.^24^

Second, our study was not designed to detect differences in seizure rates. Previously, we showed that patients carrying the risk AKT3 genotype (rs10157763, AA) exhibited more severe illness and a higher frequency of epileptic seizures compared to those with the non-risk genotypes.^11^ Similarly, sirolimus-treated mice showed a trend towards fewer seizures, although the difference did not reach difference. Seizures occurred in 17% of the mice, consistent with rates in bacterial meningitis.^6^ However, the 4-h observation intervals may have underestimated seizure incidence, limiting statistical power to detect differences. Additionally, mechanisms beyond mTORC1 hyperactivation, such as neuroinflammation and blood–brain barrier disruption, are likely involved in seizure development in pneumococcal meningitis. Elevated neuroinflammatory responses, including increased IL-1R and toll-like receptor 4 signalling and higher CSF albumin levels, may enhance neuronal hyperexcitability and potentially counteract any protective effect of sirolimus (46–49).

In conclusion, our data suggest that mTORC1 acts as a critical regulator of inflammation during pneumococcal meningitis and that its pharmacological inhibition with sirolimus can aggravate disease severity by amplifying innate immune responses. While mTORC1 inhibition has shown protective effects in other infectious contexts, our findings argue against its use as an adjunctive therapy in bacterial meningitis. These results underscore the need for context-specific therapeutic strategies that modulate the neuroinflammatory response without impairing host defence or recovery.

## Supplementary Material

fcaf460_Supplementary_Data

## Data Availability

All data generated or analysed during this study are included in this published article (and its supplementary information files).
