# Supplementary material for: mTORC1 inhibition by sirolimus as adjunctive treatment in experimental pneumococcal meningitis
Source: Brain Commun. 2025 Nov 25;8(1):fcaf460. doi: 10.1093/braincomms/fcaf460 (PMC12784197; doi:10.1093/braincomms/fcaf460)
Supplement: fcaf460_Supplementary_Data [file fcaf460_supplementary_data.pdf]

SUPPLEMENTARY MATERIALS

SUPPLEMENTARY FIGURES

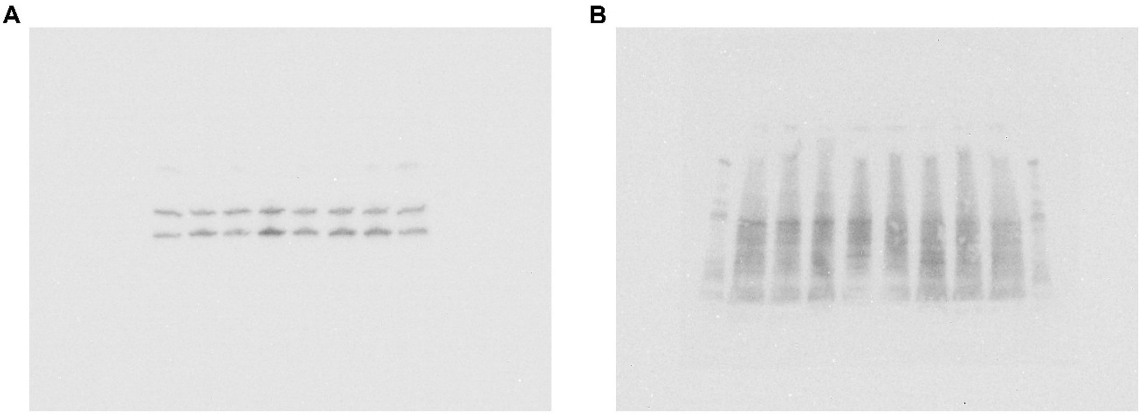

Supplementary figure 1. Full-length (uncropped) western blot images for Figure 2. (**A**) P70 S6K (upper row) and GAPDH (bottom row) in the brain of 4 control mice (left 4 lanes) with pneumococcal meningitis and 4 mice that were treated with sirolimus (right 4 lanes) at 6 hours post infection. Exposure time is 5 seconds. (**B**) Phorporylated P70 S6K in the same 8 mice as **A**. Exposure time is 15 seconds.

SUPPLEMENTARY TABLES

Supplementary table 1. Clinical severity score for murine pneumococcal meningitis

Code		Score
	**Appearance**	
	Weight loss	
1	< 5% weight loss	1
2	5-10% weight loss	2
3	11-15% weight loss	3
4	16-20% weight loss	4
5	≥20% weight loss	HEP
	Posture	
1	Slightly hunched back	1
2	Severe hunched back	2
	Coat	
1	Diminished/lack grooming	1
2	Piloerection	1
3	Combination 1+2	2
	Eyes	
1	Discharge from the eyes	1
2	Closed eyelids	1
3	Protruding eyes	1
4	Combination 1+2	2
5	Combination 1+3	2
	**Behaviour**	
	Activity	
1	Diminished activity	2
2	Inactive	3
3	Increased activity / aggressive behaviour	1
	Condition	
1	Within 5 sec to right when placed on back	2
2	Within 30 sec to right when placed on back	4
3	Inability to right after placing on back	HEP
4	Coma	HEP
	**Body function**	
	Respiration	
1	Laboured breathing	2
2	Irregular breathing,	2
3	Combination 1+2	4
	**Procedure-specific indicators**	
	Neurologic score	
1	Paresis	2
2	Coordination problem (including circling)	2
3	(Partial) seizure	2
4	Combination 1+2	4
5	Combination 1+3	4
6	Combination 2+3	4
7	Combination 1+2+3	6
8	Paralysis	HEP
9	Seizure > 5 minutes	HEP
10	≥ 2 seizures in 15 min	HEP

Abbreviations: HEP, humane endpoint

Supplementary table 2. Histopathological scoring method for murine pneumococcal meningitis

Main category	Subcategory	Score			
		0	1	2	3
Meningeal infiltration		Absent	Focal mild infiltration	Multifocal mild or focal severe infiltration	Multifocal severe infiltration
Parenchymal infiltration		Absent	Focal mild infiltration	Multifocal mild or focal severe infiltration	Multifocal severe infiltration
Vascular inflammation	Large meningeal artery inflammation<br><br>Small parenchymal vessel inflammation	Absent	Focal mild subendothelial infiltration /reactive changes	Multifocal mild subendothelial infiltration /reactive changes or focal severe vascular wall infiltration with obstruction and/or destruction of vessels	Multifocal severe vascular wall infiltration with obstruction and/or destruction of vessels
Ventriculitis		Absent	A few inflammatory cells in the ventricle	Groups of inflammatory cells in the ventricle with/without ependymal infiltration	Extension of inflammatory cells into the periventricular tissue
Hemorrhage		Absent	Focal small damage	Multifocal small or focal large damage	Multifocal large damages
Thrombosis		Absent	Focal mild with partial obstruction of vascular lumen	Multifocal mild with partial obstruction of vascular lumen or focal severe with complete obstruction of vascular lumen and destruction of vessel wall	Multifocal severe with complete obstruction of vascular lumen and destruction of vessel wall
Abscess		Absent	Focal small damage	Multifocal small or focal large damage	Multifocal large damages
